# Successful outcomes for patients with drug-resistant tuberculosis despite civil unrest and COVID-19 in Haiti

**DOI:** 10.1371/journal.pgph.0002356

**Published:** 2023-09-12

**Authors:** Stalz Charles Vilbrun, Ariadne Souroutzidis, Kathleen F. Walsh, Joshua Ellis, Colette Guiteau, Sobieskye Delva, Guy Joissaint, Patrice Joseph, Jean William Pape, Serena P. Koenig

**Affiliations:** 1 Haitian Group for the Study of Kaposi’s Sarcoma and Opportunistic Infections (GHESKIO), Port-au-Prince, Haiti; 2 The Analysis Group, Boston, Massachusetts, United States of America; 3 Center for Global Health, Weill Cornell Medicine, New York, NY, United States of America; 4 Division of General Internal Medicine, Weill Cornell Medicine, New York, NY, United States of America; 5 Harvard Medical School, Boston, Massachusetts, United States of America; 6 Division of Infectious Diseases and Division of Global Health Equity, Brigham and Women’s Hospital, Boston, Massachusetts, United States of America; Pontificia Universidad Católica de Chile: Pontificia Universidad Catolica de Chile, CHILE

## Abstract

Globally, treatment outcomes for people with multi-drug/rifampin-resistant tuberculosis (MDR/RR-TB) are sub-optimal, with MDR/RR-TB programs further weakened due to the COVID-19 pandemic, and in Haiti, by severe civil unrest. We assessed the impact of these disruptions on treatment outcomes at GHESKIO, in Port-au-Prince, Haiti. We conducted a retrospective analysis including all adults (age ≥18 years) who initiated MDR/RR-TB treatment at GHESKIO from 2010 to 2020. We assessed predictors of poor treatment outcome using multivariable logistic regression, adjusting for baseline characteristics and year of treatment. 453 patients initiated treatment for MDR/RR-TB at GHESKIO. Median age was 31 (IQR: 25, 40), 233 (51.4%) were male, and 100 (22.1%) were living with HIV. Three hundred sixty-nine patients (81.5%) achieved cure, 42 (9.3%) died, 40 (8.8%) were lost to follow-up and 2 (<1%) failed treatment. HIV status was associated with poor treatment outcome (aRR: 1.65 (95% CI: 1.09, 2.48)) but there was no difference by year of treatment initiation. Outcomes for patients with MDR/RR-TB remained outstanding, even during the COVID-19 pandemic and severe civil unrest in Haiti. We attribute this resilience in care to the adaptability of program staff and provision of economic and psychosocial support.

## Introduction

The COVID-19 pandemic has reversed years of progress in the fight to eliminate tuberculosis worldwide with re-distribution of resources from fighting TB to fighting COVID-19 exacerbating already-poor rates of treatment success [1–4]. In Haiti, a severely resource-limited country in the Caribbean, disruptions in MDR/RR-TB care have been further compounded by civil unrest [5]. Since 2018, there has been worsening widespread insecurity especially in Port-au-Prince, Haiti’s capital and largest city. The Haitian population suffers from an economic crisis, food insecurity, widespread kidnappings-for-ransom, and limited access to fuel and essential services [6, 7].

The Haitian Group for the Study of Kaposi’s Sarcoma and Opportunistic Infections (GHESKIO) is located in Port-au-Prince, Haiti, and has established a MDR/RR-TB treatment program with a focus on patient-centered care, in line with recommendations by the World Health Organization (WHO) and global health policy makers [8]. We previously published a report from this program, documenting high cure rates in the aftermath of the devastating earthquake of 2010 [9]. Since then, Haiti has been beset by numerous natural (cholera, another earthquake, COVID-19 pandemic) and man-made (political and civil unrest) challenges. Due to the long treatment duration of MDR/RR-TB, conflicts such as these could significantly derail MDR/RR-TB treatment success. Here, we evaluate predictors of poor MDR/RR-TB outcomes, including year of treatment initiation, to assess the potential impact of COVID-19 and civil unrest on GHESKIO’s MDR-TB program.

## Materials and methods

### Study site and population

GHESKIO is a non-governmental organization that has provided free HIV and TB care since 1982, and MDR/RR-TB care since 2008. GHESKIO has an inpatient MDR-TB treatment hospital with an on-site biosafety level 3 (BSL-3) laboratory. All patients ≥18 years of age with microbiologic evidence of resistance to rifampin who initiated MDR/RR-TB treatment from March 2010 to December 2020 were included in the study. Treatment outcomes were available approximately 20–24 months following treatment initiation; all patients who initiated treatment in December 2020 had outcomes available by the end of 2022.

### Description of diagnostic testing, drug regimens, and outcome definitions

Patients who presented with symptoms of TB received clinical assessment and chest radiography and provided sputum samples for mycobacterial testing including acid fast bacilli (AFB) smear and molecular testing with either Genotype MDRTB*plus* (Hain LifeScience, Nehren, Germany) or Xpert MTB/RIF (Cepheid, Sunnyvale, CA). Xpert MTB/RIF was preferentially used after May 2011. Samples with evidence of rifampin resistance by molecular testing were cultured using liquid media (BACTEC MGIT 960, Becton Dickinson, Franklin Lakes, USA) and solid media (Lowenstein-Jensen slant), and first and second-line drug susceptibility (DST) testing was conducted in accordance with the manufacturer’s recommendations, as described previously [10].

Treatment regimens changed over time, in accordance with WHO guidelines; drugs were dosed according to WHO recommendations, and regimens were adapted based on DST results (Table 1) [11–13]. Through 2011, patients were initiated on a standard regimen that included an injectable agent (kanamycin or capreomycin), fluoroquinolone, cycloserine, ethionamide and pyrazinamide. Para-amino salicylic acid (PAS) was included in the standard regimen for HIV-negative patients until 2011 (when it was replaced by high-dose isoniazid until 2014), and for persons with HIV (PWH) until 2015. Bedaquiline was first used in 2016 on an individual compassionate use basis. It was added to the standard regimen for all patients to replace the second-line injectable in 2018. The currently recommended all-oral regimen of bedaquiline, levofloxacin, linezolid, clofazimine, and pyrazinamide was implemented in April 2019 for all newly diagnosed patients. PWH initiated antiretroviral therapy (ART) within 2 weeks of starting MDR/RR-TB treatment, regardless of CD4 count, if they were not already on ART. The ART regimens prescribed included 2 nucleoside reverse transcriptase inhibitors and a backbone drug, which included a non-nucleoside reverse transcriptase inhibitor or protease inhibitor (based on the MDR-TB regimen) until December 2018, when dolutegravir became the preferred backbone drug for all patients.

**Table 1:** Changes in treatment regimens over time [14].

Year	Guiding Principle	Standard Regimen*	Number of patients per regimen
2010	Regimens should consist of at least 4 drugs with near-certain effectiveness [15].	Intensive: Lfx-Km/Cm-Z-Eto-Cs-PAS Continuation: Lfx -Z-Eto-Cs-PAS	BDQ: 0; high dose INH: 0; neither: 20
2011–2014	Regimens should consist of at least 4 drugs with near-certain effectiveness [15].	Intensive: Lfx -Km/Cm-Z-Eto-Cs-PAS (for PWH and high dose H for HIV-negative individuals Continuation: Lfx -Z-Eto-Cs-PAS/high dose H	**2011**: BDQ: 0; high dose INH: 8; neither: 5 **2012**: BDQ: 0; high dose INH: 27; neither: 8 **2013**: BDQ: 0; high dose INH: 38; neither: 6 **2014**: BDQ: 0; high dose INH: 26; neither: 21
2015	Regimens should include at least a fluoroquinolone, pyrazinamide, second-line injectable agent, ethionamide and either cycloserine or PAS [16].	Intensive: Lfx -Km/Cm-Z-Eto-Cs Continuation: Lfx-Z-Eto-Cs Bdq was used rarely for compassionate care.	BDQ: 3; high dose INH: 0; neither: 49
2016–2017	Regimens should include at least 5 effective drugs including pyrazinamide during the intensive phase [12].	Intensive: Lfx -Km/Cm-Z-Eto-Cs Continuation: Lfx-Z-Eto-Cs Bdq was used rarely for compassionate care.	**2016**: BDQ: 7; high dose INH: 0; neither: 49 **2017**: BDQ: 8; high dose INH: 0; neither: 36
2018		Intensive: Lfx-Bdq-Z-Eto-Cs Continuation: Lfx-Z-Eto-Cs	**2018**: BDQ: 9; high dose INH: 0; neither: 32
2019–2020	Regimens should include at least four effective agents at treatment initiation and at least three effective drugs should remain after bedaquiline is stopped [17].	Intensive: Lfx-Bdq-Lzd-Cfz-Z Continuation: Lfx-Lzd-Cfz-Z^	**2019**: BDQ: 37; high dose INH: 0; neither: 8 **2020**: BDQ: 36; high dose INH: 0; neither: 0

PWH: people living with HIV

Lfx: levofloxacin

km: kanamycin

Cm: capreomycin

Z: pyrazinamide

Eto: ethionamide

Cs: cycloserine

PAS: P-aminosalicyclic acid

high dose H: high dose isoniazid

Bdq: bedaquiline

Lzd: linezolid

Cfz: clofazimine

* Regimens were standardized at treatment initiation then tailored to individual patient drug susceptibility patterns as they resulted. All regimens were of longer duration.

^Lzd is discontinued after 12 months.

Sputum smear microscopy and cultures were obtained per national guidelines, which varied slightly over time; at a minimum, smears and cultures were conducted monthly during the intensive phase, then every other month, and then again monthly during the final 3 to 5 months of treatment. Culture conversion was defined as 2 consecutive negative cultures at least 30 days apart. Treatment outcomes were defined according to the 2014 WHO guidelines [16]. Cure was defined as completion of treatment without evidence of failure, and at least 3 consecutive negative cultures taken at least 30 days apart after the intensive phase (or after 8 months for regimens without a clear distinction between intensive and continuation phases). Treatment completion was defined as completion of drug regimen without failure or mycobacteriological evidence of cure. Failure was defined as prematurely ending treatment or changing 2 or more drugs due to lack of culture conversion, bacteriologic reversion, adverse drug event, or additional drug resistance. Death was defined as death due to any cause prior to treatment completion. Loss to follow-up (LTFU) was defined as treatment interruption for ≥ 2 consecutive months.

### Description of treatment location, psychosocial support, and nutritional supplementation

Through 2014, patients were hospitalized for intensive therapy, which included approximately the first 10 months, while they received an injectable medication (kanamycin or capreomycin). Initially they were hospitalized in an inpatient facility, but in January 2010 it was destroyed in a devastating earthquake, and patients were hospitalized in individual tents in a field hospital until the inpatient GHESKIO MDR-TB hospital was opened in 2015. The same year, the length of inpatient hospitalization was decreased; patients were discharged after they had converted to culture-negative (generally about 4 months). After discharge, patients were followed at home, with all doses observed through directly observed treatment (DOT) supervised by a community health worker (CHW) or a nurse (for doses that included an injectable agent). Nearly all patients were treated with long-course, standard therapy (at least 20 months in duration).

During home visits, the DOT provider queried patients about symptoms, checked vital signs, and photographed the patient taking their medication. These activities were recorded at each visit using mobile phones equipped with global positioning system (GPS) devices. Patients with severe symptoms were immediately referred for medical attention. Team meetings (physicians, nurses, and social workers) were held for patients who missed medication doses and/or visits to help identify and overcome specific barriers to adherence. Medical visits (physician and/or nurse) were scheduled as needed, and at least monthly.

Patients were offered psychosocial support through individual counseling sessions and monthly group support meetings. At the time of diagnosis, patients were requested to specify a family member or friend to provide support for the treatment period, including attending all medical visits and monthly group meetings with medical team members and other patients. At each medical visit, patients were provided with transportation subsidies and telephone cards. At each monthly group meeting, the medical team provided social support, treatment literacy counseling, and dry food rations totaling about USD $25 per patient. Each time a patient successfully completed treatment, they were awarded with a treatment completion certificate and an end-of-treatment prize (USD $200 in cash).

### Changes in treatment approaches due to civil unrest and the COVID-19 pandemic

During times of severe civil unrest that had the potential to impact travel to clinic, CHWs and nurses switched to phone-based DOT for patients who could not receive in-person DOT. This provided a safe method of communication between clinic providers and the patient. This phone-based communication was also utilized when patients were unable to travel to clinic due to safety concerns. GHESKIO staff frequently reached out to patients to help them remain engaged in care, even if this engagement was virtual. Additionally, depots were set up in safer areas of Port-au-Prince, where patients were able to obtain their medications if they felt that travel to GHESKIO was too dangerous, and patients were provided with several weeks’ reserve supply of medications to facilitate adherence if they were unable to receive medications.

**Fig 1:**
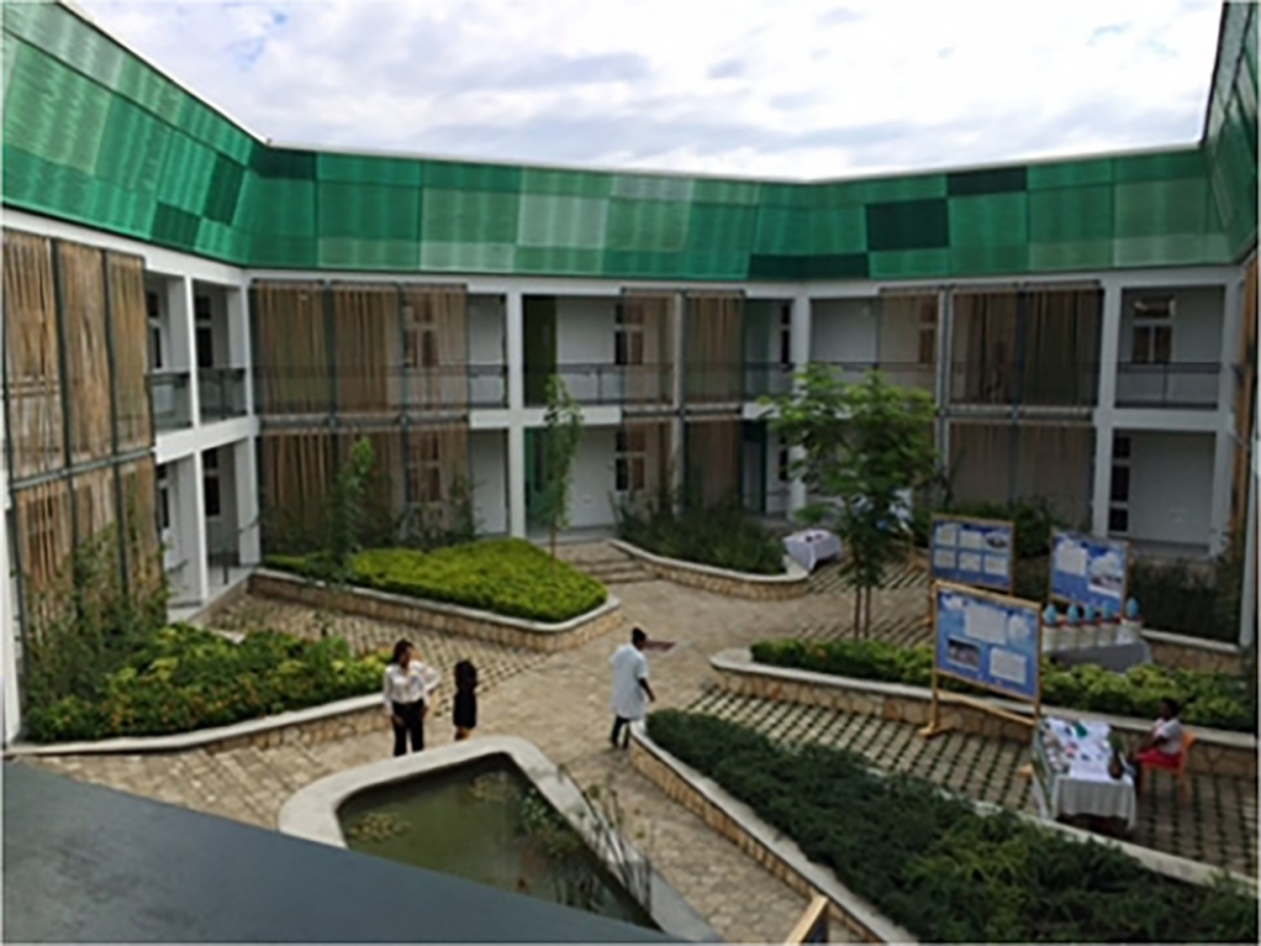
GHESKIO’s inpatient facility for treatment of multi-drug/Rifampin-resistant tuberculosis. Photograph taken by and provided with permission by Dr. Warren D. Johnson.

GHESKIO’s inpatient MDR-TB facility was designed by the Model of Architecture Serving Society (MASS) Design Group, a non-profit architectural firm, with a focus on minimizing the risk of TB transmission [18]. The hospital has outdoor community space, outdoor corridors and consultation areas, permeable soffits and metal louvres to pull fresh air through the inpatient units (Fig 1). It was therefore ideally adapted for the prevention of all airborne infections, including SARS-CoV-2. All MDR/RR-TB patients were tested for COVID-19 at time of admission. All staff used protective personal equipment (PPE) as clinically appropriate, and monthly in-person support meetings were stopped to prevent SARS-CoV-2 transmission. Additionally, GHESKIO initiated an intensive educational campaign among community members and patients to combat misinformation and stigmatization associated with SARS-CoV-2 infection.

### Statistical analyses

Demographic and clinical data were extracted from the GHESKIO electronic medical record, the GHESKIO laboratory database and hard-copy paper charts. Year of treatment initiation was categorized as a binary variable, based on whether it occurred prior to 2018, or during or after 2018, as 2018 marked the period when civil unrest increasingly affected day-to-day activities in Port-au-Prince. Treatment outcomes were dichotomized into successful (cure, treatment completion,) and unsuccessful (treatment failure, LTFU, or death). Log-binomial regression was used to determine predictors of unsuccessful treatment as a composite outcome and death and LTFU as individual outcomes. Predictors were considered if there was a sample size of at least 10 patients. Variables with a p-value of <0.20 or a relative risk (RR) of <0.80 or >1.2 in the univariable model were considered for inclusion in the multivariable model. All analyses were conducted with R software (Version 4.0.5).

### Ethical approval

It was not feasible to obtain informed consent due to the retrospective nature of the research. This study used routinely collected data stored in the medical record for patients receiving care at GHESKIO. The study was approved by the institutional review boards of GHESKIO, Weill Cornell Medicine and Brigham and Women’s Hospital.

## Results

From 2010 to 2020, 453 adult patients were treated for MDR/RR-TB at GHESKIO; of these 331 (73.1%) initiated treatment prior to 2018. The median age was 31 (interquartile range [IQR]: 25, 40) and 233 (51.4%) patients were male (Table 2). A total of 100 patients (22.1%) were living with HIV.

**Table 2:** Patient characteristics.

	Total Cohort (n = 453)
Age (years)–median (IQR)	31 (25, 40)
Male sex–no. (%)	233 (51.4)
Lives on <$US 1 per day	426 (94.0)
Education at primary level or lower–no. (%)	191 (42.2)
Living with HIV–no. (%)	100 (22)
Diabetes–no. (%)	10 (2.2)
Past TB Treatment–no. (%)	306 (67.5)
Treatment before 2018 –no. (%)	331 (73.1)
Resistant to fluoroquinolone and/or second-line injectable–no. (%)	12 (2.6)

**Fig 2:**
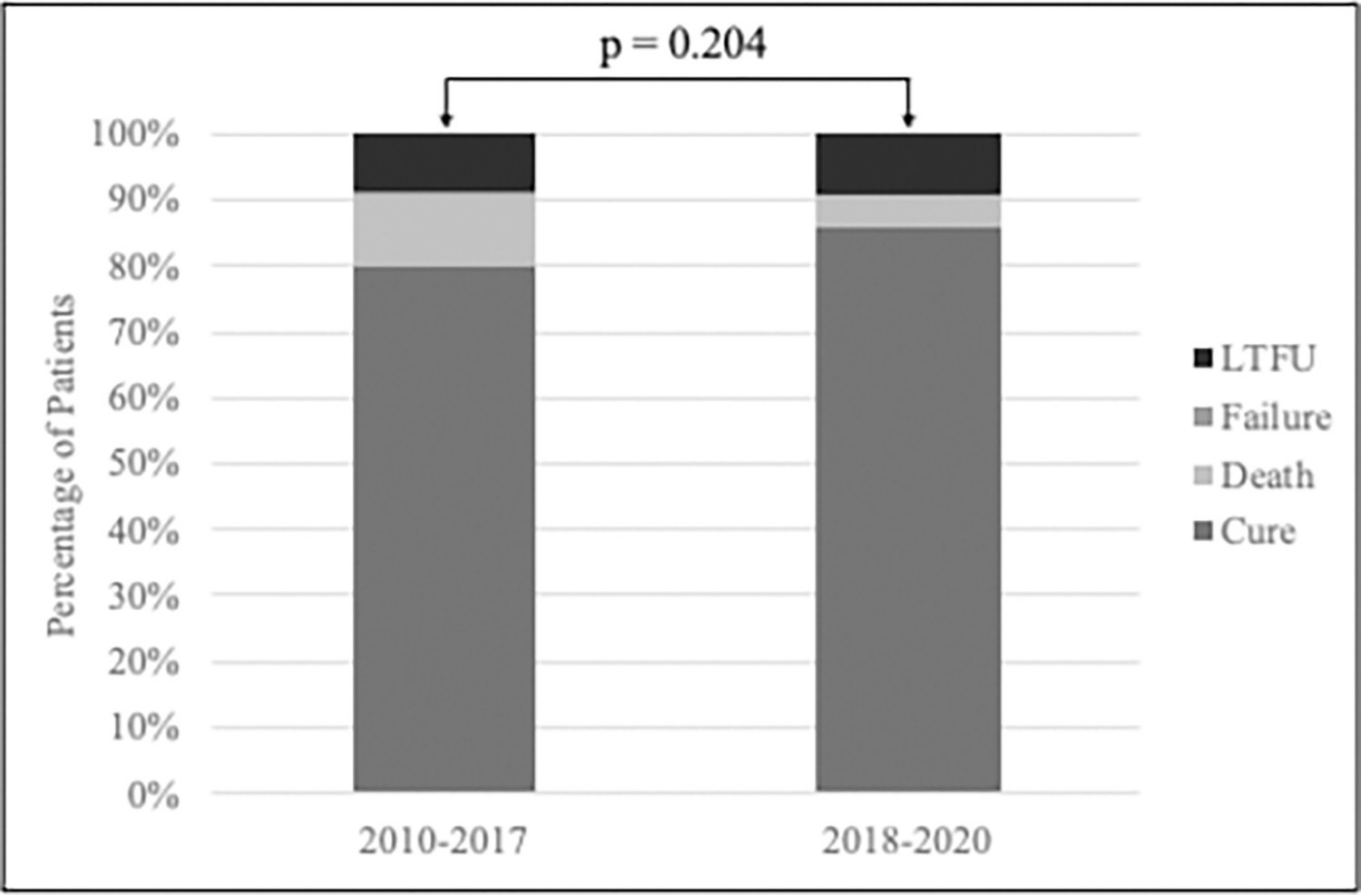
Treatment outcomes, 2010–2017 compared to 2018–2020.

**Fig 3:**
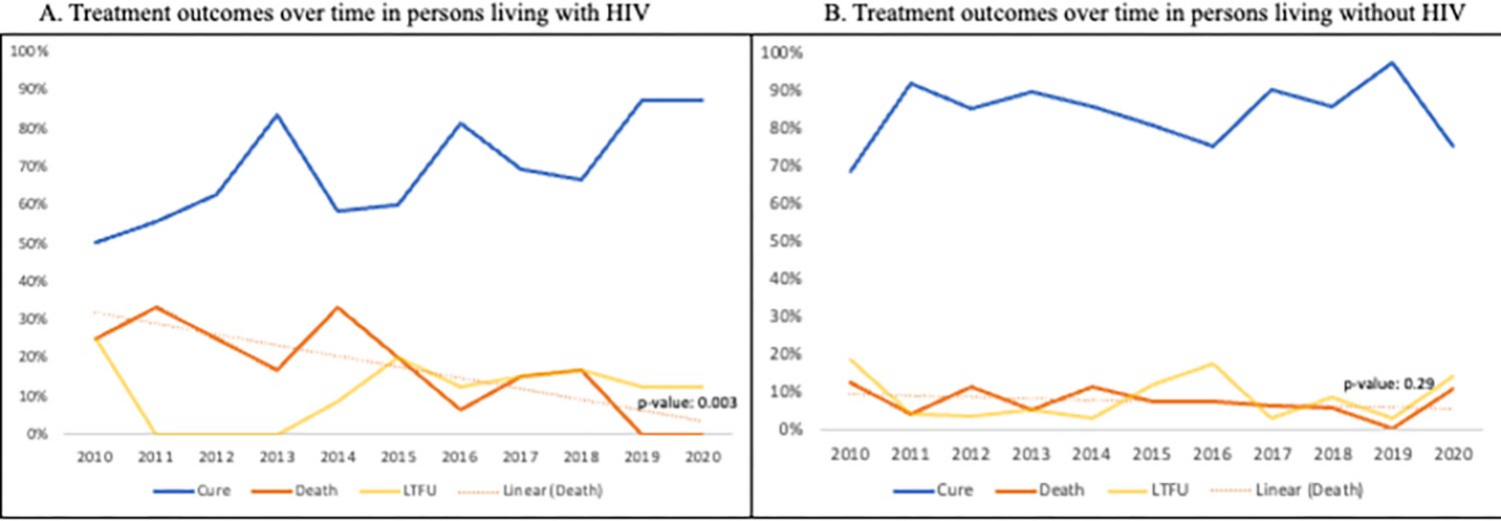
A. Treatment outcomes over time in persons living with HIV. B. Treatment outcomes over time in HIV-negative persons.

In total, 369 (81.5%) patients have achieved cure, 42 (9.3%) have died, 40 (8.8%) have been loss to follow-up (LTFU) and 2 (<1%) experienced treatment failure (Table 3). Among the total cohort, the median time to death was 113 days (IQR: 40, 300); among PWH, the median time to death was 104 days (IQR: 42, 211). Among patients treated prior to 2018, the proportion with cure, death, LTFU, and failure were 79.8%, 10.9%, 8.8%, and 0.6%, respectively. Among those treated from 2018 or later, these proportions were 86.1%, 4.9%, 9.0%, and 0, respectively. As illustrated in Fig 2, there was no difference in outcomes between these two time periods (p = 0.204). Treatment success rates were higher among HIV-negative patients (84.7% vs. 70.0%) and PWH were more likely to die than those without HIV (17.0% vs. 7.1%). Treatment outcomes significantly improved over time for PWH (Fig 3A) whereas successful treatment outcomes for those without HIV remained consistently high over time (Fig 3B).

**Table 3:** MDR/RR-TB treatment outcomes, stratified by HIV status.

	HIV-negative (n = 353, %)	PLW (n = 100, %)	Total Cohort (n = 453, %)
Cure/Treatment completion–no. (%)	299 (84.7)	70 (70.0)	369 (81.5)
Death–no. (%)	25 (7.1)	17 (17.0)	42 (9.3)
Lost to follow-up–no. (%)	29 (8.2)	11 (11.0)	40 (8.8)
Failure–no. (%)	0 (0)	2 (2.0)	2 (0.4)

In the univariate analysis examining poor treatment outcome as a composite (treatment failure, LTFU, or death combined), positive HIV status (RR 1.96; 95% CI: 1.31, 2.86) was associated with unsuccessful treatment outcome; receiving high-dose INH (RR: 0.54; 95% CI: 0.28, 0.93) was associated with a successful treatment outcome. The multivariable analysis yielded similar results, with an adjusted RR (aRR) 1.65 (95% CI: 1.09, 2.48) for positive HIV status. For those receiving high-dose INH, the aRR 0.56 (95% CI: 0.28, 1.02) trended towards significance. Resistance to fluoroquinolone or second-line injectable was not included in the adjusted model given the small sample size (Table 4). There was no difference in risk of unsuccessful treatment outcome by year of treatment initiation (aOR for treatment prior to 2018: 1.60; 95% CI: 0.99, 2.72). We also conducted univariate analyses for the specific outcomes of death and LTFU. HIV status was significantly associated with death (RR 2.40 [1.33, 4.23], p = 0.003). There were no other significant findings in the individual univariate model.

**Table 4:** Predictors of poor treatment outcomes.

Predictor	Unadjusted Relative Risk			Adjusted Relative Risk	
	RR (95% CI)	p-value	RR (95% CI)		p-value
Age	1.00 (0.98, 1.02)	0.958	-		
Male sex	0.99 (0.67, 1.46)	0.960	-		
Education primary school or lower	1.01 (0.67, 1.50)	0.954	-		
Living with HIV	1.96 (1.31, 2.86)	0.001	1.65 (1.09, 2.48)		0.016
Treatment before 2018	1.45 (0.92, 2.46)	0.135	1.60 (0.99, 2.72)		0.068
Received high-dose INH	0.54 (0.28, 0.93)	0.041	0.56 (0.28, 1.02)		0.074
Received BDQ	0.77 (0.44, 1.24)	0.311			

Diabetes mellitus, prior TB treatment, resistance to fluoroquinolone or injectable agent and income all had sample sizes < 10 individuals and were not included in the multivariate analysis.

## Discussion

Our results demonstrate that it is possible to maintain superb MDR/RR-TB outcomes in a resource-poor setting with tremendous barriers to accessible care. We attribute the resilience of GHESKIO’s MDR/RR-TB program to three key factors: adaptable staff, economic support, and psychosocial support. GHESKIO’s approach to TB care is not new–Dr. Paul Farmer, co-founder of Partners in Health (PIH), advocated for a biosocial approach to TB care for decades. This approach is based on the “5 S’s”: staff, stuff, space, systems and social support which serve as the foundation of healthcare strengthening at PIH [19, 20]. GHESKIO provides one more example of the successful application of this approach.

GHESKIO’s staff routinely adapted their provision of care to current events. When necessary, DOT was performed by cell phone with patients photographing themselves taking medicine and sharing these pictures with staff. Medication depots were created in safe neighborhoods so that patients could obtain their medications without putting themselves at risk. Additionally, patients were provided with several weeks’ reserve supply of medications to facilitate adherence if they were unable to receive medications. Finally, GHESKIO deployed CHWs to surrounding communities to provide information regarding COVID-19, thus combatting the misinformation and stigmatization experienced in Haiti and globally [21, 22].

The COVID-19 pandemic, in particular, highlights the importance of GHESKIO’s work and its ability to adapt. Globally, COVID-19 has negatively impacted HIV and TB care, maternal and child health and noncommunicable disease management [1–4, 23–26]. This was true in Haiti as well, with increases in childhood cases of diphtheria from decreased vaccinations, increased malaria incidence and increased maternal deaths. All of these negative health indicators were likely caused in part from the inability to access care in the midst of the pandemic, and in Haiti especially, from increased civil unrest. GHESKIO’s ability to provide continuous MDR-TB services, despite numerous obstacles, provides a model of care that can be utilized in diverse resource-poor settings worldwide and which can be adapted to different diseases.

Patients with MDR/RR-TB face economic barriers which, if not addressed, contribute to poor treatment outcomes [27–29]. GHESKIO provided transportation waivers and nutritional support to all patients throughout treatment as well as a monetary prize upon treatment completion. Similar interventions have been shown to be successful in other low-resource settings [27, 30]. For example, in the Democratic Republic of Congo, a MDR/RR-TB treatment program provided transportation subsidies and nutritional support and maintained a LTFU rate of only 5%, compared to the global average of 15% [31, 32]. The low LTFU and failure rates seen in our cohort are likely due in part to GHESKIO’s efforts to offset patients’ financial burden.

Lack of social support has been associated with LTFU among persons with MDR/RR-TB [33]. GHESKIO’s patients were offered individual counseling and monthly group support sessions, which gave patients and their families an opportunity to identify, understand and overcome the significant impact of this potentially stigmatizing disease on their lives. This is similar to a programmatic intervention in Peru among patients with MDR/RR-TB who engaged with bi-monthly support groups wherein only 3.5% were LTFU [34]. In a meta-analysis on the effectiveness of social support on treatment outcomes for drug-resistant TB, programs that offered social support along with material support had 2.6 greater odds of treatment success (95% CI: 1.8, 3.7) and reduced LTFU (OR: 0.17; 95% CI: 0.05, 0.55) [35].

In our cohort, we found that mortality rates were higher in PWH. This has been reported in other studies as well [36, 37]. It is noteworthy that mortality among PWH has decreased over time in our cohort, which we attribute in part to the recent availability of new and re-purposed medications that are more effective against MDR/RR-TB, and potentially the adoption of dolutegravir into most ART regimens as well.

Our findings are limited in part due to changing regimens over time, which makes it challenging to determine to what extent specific regimens may have affected our outcomes. The study was conducted in a single treatment facility which may not be representative of the entire country. The cohort was limited to those patients who initiated treatment prior to or in 2020, and thus had treatment outcomes available at the time of publication of this manuscript. This may not capture the long-term impacts of COVID-19. Providing economic and social support does require significant commitment and resources which may impact the generalizability of our outcomes to other regions. Haiti is a low resource country, however, which highlights that the distribution of resources to individuals with TB is important and achievable.

## Conclusion

We report ongoing outstanding treatment outcomes for MDR/RR-TB in Port-au-Prince, Haiti, in spite of major civil unrest and disruptions from the COVID-19 pandemic. We attribute this success to the provision of adaptive services and economic and social support.

## Supporting information

S1 Data Manuscript data submission. *This is the dataset upon which the data presented in this manuscript is based. Variables: Patient_ID: unique anonymous identifier for each patient; Tx initiation year: The time frame during which the patient received treatment; Age_cat: age at time of TB diagnosis, grouped into 10-year groups; Sex: self-reported sex of the patient; HIV: HIV serostatus at time of diagnosis; Education: highest educational level obtained by the patient (self-reported), grouped into a binary variable; Income: self-reported daily income of the patient, grouped into a binary variable; Outcome: TB treatment outcome based on World Health Organization definitions; preXDR: detailing whether a patient had resistance to a second-line injectable agent or fluoroquinolone, with 0 = no, 1 = yes; Tx Cohort: the describes the treatment cohort the patient was in for the study’s primary analysis; regimen with high dose INH, a regimen with BDQ, or a regimen containing neither.
(XLSX)
